# Relação entre Fibrose Hepática Decorrente da Doença Hepática Gordurosa Associada à Disfunção Metabólica e Aterosclerose Subclínica

**DOI:** 10.36660/abc.20240856

**Published:** 2025-08-20

**Authors:** Daniele Araujo de Azeredo Coutinho, Jenaine Rosa Godinho, Raphael Carreiro Moura, Rogério Martins Oliveira, Hevila Faria Passos, Jordana de Paula Felipe Mendes, Carlos Roberto Moraes de Andrade, Luis Guillermo Coca-Velarde, Maria Auxiliadora Nogueira Saad, Priscila Pollo Flores, Debora Vieira Soares

**Affiliations:** 1 Universidade Federal Fluminense Niterói RJ Brasil Universidade Federal Fluminense, Niterói, RJ – Brasil; 2 Universidade Federal Fluminense Hospital Universitário Antônio Pedro Niterói RJ Brasil Universidade Federal Fluminense - Hospital Universitário Antônio Pedro, Niterói, RJ – Brasil

**Keywords:** Aterosclerose, Diabetes Mellitus Tipo 2, Cirrose Hepática, Fatores de Risco de Doenças Cardíacas

## Abstract

**Fundamento:**

A doença hepática esteatótica associada à disfunção metabólica (DHEADM) é a enfermidade hepática mais prevalente no mundo. A DHEADM pode progredir para fibrose e complicações associadas, sendo a doença cardiovascular a principal causa de morte nesses indivíduos. Marcadores de aterosclerose carotídea são capazes de predizer desfechos cardiovasculares, o que ressalta a importância de sua associação com a fibrose hepática.

**Objetivo:**

Avaliar a relação entre fibrose hepática na DHEADM e aterosclerose subclínica, por meio da análise da espessura médio-intimal da carótida (EMIC), da idade vascular (IV) e da presença de placas ateroscleróticas.

**Métodos:**

Estudo prospectivo conduzido com participantes sob risco para DHEADM. A esteatose e a fibrose hepáticas foram avaliadas por ultrassonografia e elastografia hepática, com uso do parâmetro de atenuação controlada. A aterosclerose carotídea foi analisada por meio de ultrassonografia de carótidas, medições da EMIC e da IV. Adotou-se nível de significância de 5% (p < 0,05).

**Resultados:**

Foram incluídos 114 participantes, com idade mediana de 64 anos (IIQ: 55–68), sendo 96 (84%) do sexo feminino. A esteatose foi identificada em 99 participantes (86,8%) e a fibrose em 31 (27,2%). Placas ateroscleróticas estavam presentes em 33 indivíduos (28,9%), sem diferença significativa na frequência entre os grupos. No entanto, o grupo com fibrose apresentou EMIC mais elevada e IV aumentada. Em uma análise de subgrupo com 85 participantes com diabetes mellitus tipo 2 (DM2), 27 (31,8%) apresentavam fibrose. Esses indivíduos apresentaram EMIC superior (0,742 mm vs. 0,653 mm; p < 0,05) e IV nove anos acima da idade cronológica, quando comparados aos indivíduos sem fibrose (p < 0,05).

**Conclusão:**

Este estudo demonstra que indivíduos com DHEADM e fibrose hepática apresentam maior envelhecimento vascular e risco cardiovascular aumentado, refletidos por elevações na EMIC e na IV.

## Graphical abstract

**Figura Central:**
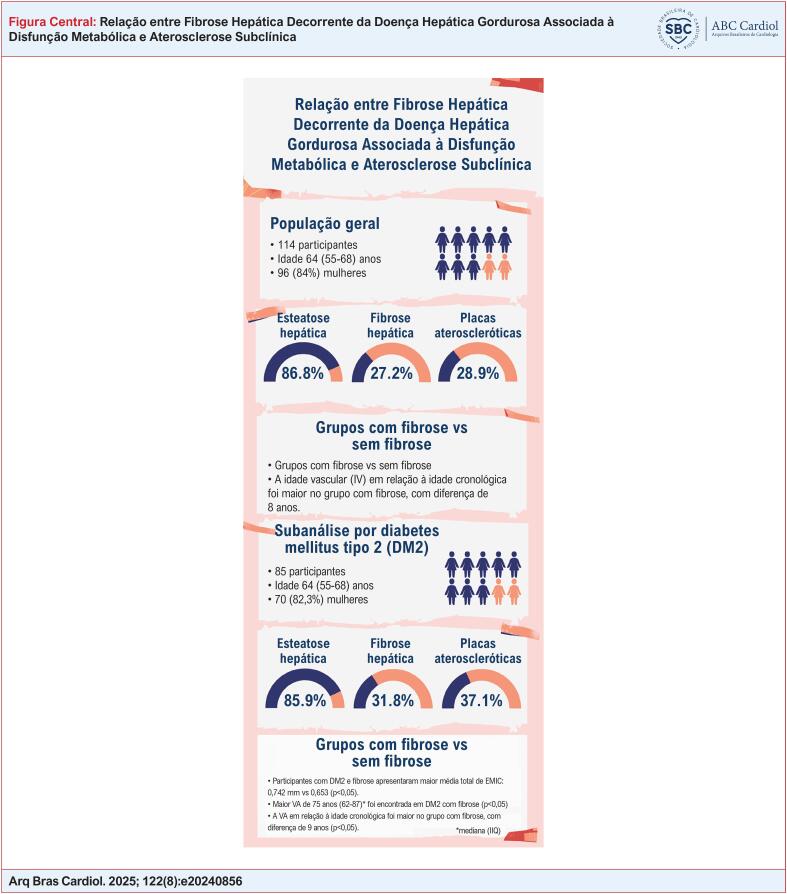
Relação entre Fibrose Hepática Decorrente da Doença Hepática Gordurosa Associada à Disfunção Metabólica e Aterosclerose Subclínica

## Introdução

A doença hepática esteatótica associada à disfunção metabólica (DHEADM)^1^ é uma condição altamente prevalente, afetando aproximadamente 30% da população mundial e até 44% na América Latina.^2^ A DHEADM abrange um espectro que vai desde a esteatose simples até a esteato-hepatite (DHEADM com inflamação ativa, anteriormente denominada MASH), fibrose e, por fim, cirrose hepática. Na população geral, cerca de 10% dos indivíduos com DHEADM evoluem para esteato-hepatite, fibrose e doença hepática avançada. No entanto, essa proporção é significativamente maior entre pessoas com diabetes mellitus tipo 2 (DM2) e obesidade.^1 , 3 , 4^

A mortalidade geral é elevada em indivíduos com DHEADM e fibrose, sendo a doença cardiovascular (DCV) a principal causa de óbito.^3 , 5^ Nos casos de fibrose avançada e cirrose, a mortalidade por causas hepáticas torna-se mais proeminente.^3^

Os principais fatores de risco metabólicos para a DHEADM estão associados à resistência à insulina, síndrome metabólica (SM), obesidade visceral, diabetes mellitus e dislipidemia aterogênica.^1 , 3^ Embora parte do risco cardiovascular possa ser atribuída a essas comorbidades, o diagnóstico de DHEADM com fibrose está associado, de forma independente, ao aumento da mortalidade cardiovascular.^6 , 7^

Estudos prévios demonstraram que a medição da espessura médio-intimal da carótida (EMIC) por meio da ultrassonografia carotídea é um preditor independente de risco cardiovascular. A EMIC está associada à probabilidade de ocorrência de eventos isquêmicos coronarianos e cerebrovasculares futuros, mesmo em indivíduos assintomáticos.^8^ Por sua vez, a DHEADM e a presença de fibrose hepática também podem atuar como preditores potenciais de aterosclerose subclínica.^9^ Diversos estudos relataram uma associação entre DCV subclínica e fibrose hepática decorrente da DHEADM.^9 , 10^

O objetivo do presente estudo foi avaliar a relação entre a fibrose hepática na DHEADM e a aterosclerose subclínica, por meio da análise da EMIC, do cálculo da idade vascular e da determinação da frequência de placas ateroscleróticas.

## Métodos

Trata-se de um estudo prospectivo, observacional, com análise de dados transversais. Os participantes foram recrutados no ambulatório de Endocrinologia de um hospital universitário entre março de 2020 e agosto de 2021. O recrutamento foi interrompido em razão da pandemia de COVID-19. O estudo foi aprovado pelo Comitê de Ética em Pesquisa local, sob o protocolo CAAE: 51731721.7.0000.5243. Todos os participantes assinaram o termo de consentimento livre e esclarecido.

Os critérios de inclusão foram idade superior a 18 anos e presença de fatores de risco para DHEADM, incluindo pré-diabetes, DM2, obesidade e SM. A SM foi definida segundo os critérios da Federação Internacional de Diabetes (IDF), que exigem obesidade central (circunferência da cintura > 90 cm em homens e > 80 cm em mulheres), além de dois ou mais dos seguintes critérios: triglicerídeos ≥ 150 mg/dl ou uso de fármacos hipolipemiantes; colesterol HDL < 40 mg/dl em homens e < 50 mg/dl em mulheres ou uso de fármacos hipolipemiantes; pressão arterial sistólica ≥ 130 mmHg e/ou pressão arterial diastólica ≥ 85 mmHg ou uso de medicação anti-hipertensiva; glicemia de jejum ≥ 100 mg/dl ou diagnóstico prévio de DM2.^10^

Os critérios de exclusão incluíram histórico atual ou pregresso de consumo de álcool > 14 g/dia para mulheres e > 20 g/dia para homens, câncer, uso de corticosteroides sistêmicos ou androgênios, terapia hormonal para menopausa, histórico de cirurgia bariátrica, gestação e doença renal crônica com taxa de filtração glomerular (TFG) < 60 ml/min/1,73 m^2^, calculada por meio da equação da Chronic Kidney Disease Epidemiology Collaboration (CKD-EPI).^11^

Com relação às doenças hepáticas, foram excluídos indivíduos com hepatites virais (por exemplo, antígeno de superfície da hepatite B, anticorpo contra o antígeno de superfície da hepatite B, anticorpo contra o antígeno core da hepatite B e anticorpo contra o vírus da hepatite C) e hepatite autoimune. A triagem para hepatite autoimune foi realizada por meio da dosagem de IgG e de autoanticorpos antinucleares. Em pacientes com elevação de enzimas hepáticas e pelo menos um resultado positivo ou alterado, foram realizados testes adicionais, incluindo a dosagem de anticorpo antimusculatura lisa e de anticorpo antimicrossoma fígado-rim tipo 1. Indivíduos com carcinoma hepatocelular, cirrose classe C de Child-Pugh ou cirrose descompensada também foram excluídos.

Os participantes foram categorizados em grupos com e sem fibrose hepática. A presença de fibrose hepática foi definida como rigidez hepática ≥ 7,1 kPa, valor correspondente ao estágio F2 de fibrose na biópsia hepática, de acordo com o sistema de escore Metavir.^12 , 13^ Também foi realizada uma análise de subgrupo com os participantes com DM2.

As características demográficas, fatores de estilo de vida, uso de medicações e histórico prévio de DCV — incluindo acidente vascular cerebral, doença arterial coronariana e doença arterial periférica — foram obtidos por meio de prontuários médicos.

As avaliações clínicas incluíram a aferição do peso corporal em balança antropométrica Filizola^®^ calibrada (São Paulo, SP, Brasil), certificada pelo Instituto Nacional de Metrologia, Normalização e Qualidade Industrial (Inmetro). A estatura foi medida com estadiômetro, com o participante em posição ortostática. A circunferência da cintura foi medida com fita métrica inelástica SANNY (São Bernardo do Campo, SP, Brasil), no ponto médio entre a crista ilíaca e a última costela, com o participante em pé e ao final de uma expiração normal.^14^ A circunferência do pescoço (CP) foi aferida na base do pescoço, logo abaixo da proeminência laríngea, com o participante em pé, olhando para frente e com os ombros relaxados. Considerou-se CP elevada se ≥ 35,5 cm para homens e ≥ 32 cm para mulheres.^15^ A razão cintura-estatura (RCEst) foi considerada elevada se ≥ 0,5. O índice de massa corporal (IMC) foi calculado, e definiu-se obesidade como IMC ≥ 30 kg/m^2^, segundo os critérios da Organização Mundial da Saúde (OMS).^14^

A coleta de sangue foi realizada após jejum noturno de 12 horas. Os seguintes exames laboratoriais foram realizados por meio do método imunoturbidimétrico: glicemia de jejum, colesterol total (CT), colesterol de lipoproteína de alta densidade (HDL-c), triglicerídeos (TG), enzimas hepáticas, ureia, creatinina, bilirrubinas, proteína C reativa ultrassensível e ácido úrico. Ferritina, hormônio estimulador da tireoide (TSH) e T4 livre foram dosados por imunensaio de quimioluminescência, utilizando kits comerciais do sistema Alinity S (São Paulo, Brasil). O colesterol de lipoproteína de baixa densidade (LDL-c) foi estimado por meio da equação de Friedewald. A contagem de plaquetas foi determinada pelo sistema de análise celular Unicel^®^ DxH 800, Coulter^®^.

A esteatose hepática foi avaliada por meio de ultrassonografia hepática e elastografia transitória, incluindo a medição do parâmetro de atenuação controlada (CAP), utilizando o modelo FibroScan^®^ Touch 502. Todos os exames foram realizados após jejum de 4 horas, por um mesmo operador experiente. A esteatose foi classificada como leve, moderada ou grave, segundo a classificação de Saadeh.^16^ Também foi utilizado o Índice de Fígado Gorduroso (FLI-score) para estimar a probabilidade de esteato-hepatite, considerando-se FLI-score ≥ 4 pontos como indicativo de alta probabilidade.^17 , 18^

A quantificação da esteatose pelo CAP foi categorizada da seguinte forma: < 236 dB/m indicou ausência de esteatose ou teor de gordura hepática < 5%; 236–279 dB/m indicou esteatose leve (5–33%); 279–302 dB/m indicou esteatose moderada (33–66%); e ≥ 302 dB/m indicou esteatose acentuada (> 66%).^19^

A fibrose hepática foi avaliada por meio de medições da rigidez hepática utilizando elastografia transitória unidimensional (FibroScan^®^, Echosens, França), com sondas M ou XL selecionadas conforme o biotipo do participante, e por elastografia hepática bidimensional com pulso de força de radiação acústica (Canon^®^ Aplio i800, Japão). Os exames foram realizados após jejum de 4 horas, pelo mesmo operador experiente. A classificação da fibrose foi inferida com base no escore semiquantitativo METAVIR, de acordo com os seguintes valores de rigidez hepática: F0 ou F1: < 7,1 kPa; F2: ≥ 7,1 kPa e ≤ 9,5 kPa; F3: > 9,5 kPa e ≤ 12,5 kPa; F4: > 12,5 kPa.^20 , 21^

As avaliações ultrassonográficas das carótidas foram realizadas pelo mesmo examinador experiente, que desconhecia a alocação dos participantes nos grupos do estudo. A EMIC foi mensurada digitalmente com o sistema de ultrassonografia EPIQ7 (Philips^®^), equipado com transdutor linear de 12–13 MHz. Com o uso do software Qlab (Philips^®^), a EMIC foi aferida em milímetros, com precisão de três casas decimais. As medições seguiram a técnica padronizada pelo Departamento de Imagem Cardiovascular da Sociedade Brasileira de Cardiologia.^22^

Foi realizada uma varredura transversal das artérias carótidas e de seus ramos extracranianos, bilateralmente, para identificação de placas visíveis. Em seguida, com o transdutor posicionado longitudinalmente ao longo da artéria carótida e utilizando o programa semiautomático de detecção da EMIC, foram obtidas as medidas da EMIC a partir da parede distal da artéria carótida comum, nos últimos 10 mm proximais ao bulbo carotídeo, em três ângulos distintos: posterior, lateral e anterior. Isso resultou em seis medições — três no lado direito e três no lado esquerdo. Foram então calculadas as médias para o lado esquerdo (ML EMIC), lado direito (MR EMIC) e ambos os lados combinados (MT EMIC).

Considerou-se EMIC aumentada quando o valor ultrapassava o percentil 75 em pelo menos um dos lados. Os valores de referência foram baseados nas diretrizes de ultrassonografia vascular do Departamento de Imagem Cardiovascular da Sociedade Brasileira de Cardiologia.^22^ Foram aplicadas diferentes tabelas conforme a faixa etária dos participantes: para indivíduos com menos de 40 anos, utilizou-se a tabela do estudo CAPS, que não diferencia por etnia; para aqueles entre 40 e 65 anos, foi aplicada a tabela do estudo ELSA-Brasil, com valores de referência para indivíduos de etnia parda, refletindo a população estudada; para participantes com mais de 65 anos, utilizou-se a tabela do estudo MESA para população hispânica.^22^

Para o cálculo da IV, utilizou-se a média da EMIC de cada lado. A IV foi definida como a idade na qual o valor da EMIC correspondia ao percentil 50 bilateralmente. Nos casos em que a IV diferia entre os lados direito e esquerdo, considerou-se o maior valor.^22^

A estratificação do risco cardiovascular foi realizada por meio do escore proposto pela Sociedade Brasileira de Cardiologia, que classifica o risco em quatro categorias: baixo, moderado, alto e muito alto.^23^

### Análise estatística

Os resultados foram apresentados como mediana e intervalo interquartil (p25–p75) para variáveis contínuas com distribuição não normal, média ± desvio padrão para variáveis contínuas com distribuição normal, e frequências absolutas (n) e relativas (%) para variáveis categóricas. O teste de Shapiro-Wilk foi utilizado para avaliar a normalidade dos dados numéricos.

Para comparações entre grupos, utilizou-se o teste t para amostras independentes nas variáveis com distribuição normal, e o teste *U* de Mann-Whitney para aquelas com distribuição não normal. As variáveis categóricas foram analisadas por meio do teste exato de Fisher, conforme as frequências esperadas.

Após a verificação da normalidade, a correlação de Spearman foi aplicada para avaliar a relação entre variáveis contínuas. Valores de p < 0,05 foram considerados estatisticamente significativos. Todas as análises foram realizadas no software R, versão 3.6.1 para Windows.

## Resultados

Os principais achados deste estudo estão ilustrados na Figura Central, que resume visualmente os dados mais relevantes apresentados neste artigo.

Foram incluídos 114 participantes, dos quais 96 (84%) eram mulheres. A mediana de idade foi de 64 anos. A Tabela 1 apresenta as características basais da população do estudo. Não foram observadas diferenças significativas entre os grupos com e sem fibrose hepática em relação à idade, sexo, etnia ou comorbidades associadas.

**Tabela 1:** Dados gerais e comorbidades

Variável	População geral (n=114)	Sem fibrose (n=82)	Com fibrose (n=31)	Valor p	DM2 sem fibrose (n=58)	DM2 com fibrose (n=58)	Valor p
**Idade (anos)**	64 (55-68)	63 (55-68)	65 (58-69)	0,2197	64 (57-68)	66 (62-70)	0,0829
**Sexo feminino (n, %)**	96 (84)	67 (81,7)	28 (90,3)	0,4074	45 (77,6)	25 (92,6)	0,1286
**Hipertensão (n, %)**	101 (88,6)	70 (85,4)	30 (96,8)	0,1721	51 (87,9)	27 (100)	0,0916
**DCV (n, %)**	14 (12,3)	11 (13,4)	3 (9,7)	0,8274	8 (13,8)	3 (11,1)	0,0777
**DAP (n, %)**	7 (6,1)	3 (3,7)	4 (12,9)	0,8273	2 (3,5)	4 (14,8)	0,1471
**Dislipidemia (n, %)**	96 (84,2)	70 (85,4)	25 (80,6)	0,7461	52 (89,7)	23 (85,2)	0,7189
**Pré-DM2 (n, %)**	19 (16,7)	17 (20,7)	2 (6,5)	0,1262	-	-	-
**DM2 (n, %)**	86 (75,4)	58 (70,7)	27 (87,1)	0,1203	-	-	-
**Sobrepeso (n, %)**	26 (22,8)	22 (26,8)	4 (12,9)	0,1872	17 (29,3)	3 (11,1)	0,0985
**Obesidade (n, %)**	65 (57,0)	45 (54,9)	20 (64,5)	0,1805	27 (46,6)	18 (66,7)	0,1047
**SM IDF (n, %)**	71 (62,3)	50 (61)	20 (64,5)	0,8976	35 (60,3)	19 (70,4)	0,4702
**Uso de estatina (n, %)**	96 (85)	69 (85,2)	26 (83,9)	1	50 (86,2)	24 (88,9)	1
**Menopausa (n, %)**	85 (90,4)	59 (89,4)	25 (92,6)	0,9305	40 (90,9)	23 (95,8)	0,6493
**Tabagismo (n, %)**				0,9931			0,8234
Nunca fumou	76 (66,7)	55 (67,1)	21 (67,7)		39 (67,2)	17 (63)	
Fumante atual	4 (3,5)	3 (3,7)	1 (3,2)		1 (1,7)	1 (3,7)	
Ex-fumante	34 (29,8)	24 (29,3)	9 (29,0)		18 (31,0)	9 (33,3)	
**Atividade física (n, %)**							
Sedentário	81 (71,1)	55 (67,1)	25 (80,6)	0,1270	37 (63,8)	23 (85,2)	0,0585
>150 min/sem	22 (19,3)	18 (22)	4 (12,9)		13 (22,4)	2 (7,4)	
< 150 min/sem	10 (8,8)	9 (11)	1 (3,2)		8 (13,8)	1 (3,7)	
**Microalbuminúria (n, %)**	9 (7,9)	4 (4,9)	5 (16,1)	0,1137	4 (6,9)	5 (18,5)	0,1351
**Retinopatias (n, %)**							
RCV (n, %):	4 (3,5)	2 (2,4)	2 (6,5)	0,5861	0 (0)	0 (0)	0,9468
Baixo	7 (6,1)	4 (4,9)	3 (9,7)		3 (8,6)	1 (3,7)	
Moderado	89 (78,1)	65 (79,3)	23 (74,2)		47 (81,0)	23 (85,2)	
Alto	12 (10,5)	9 (11)	3 (9,7)		6 (10,3)	3 (11,1)	
Muito alto							

Os valores são expressos como média e desvio padrão para dados com distribuição normal, e como mediana e intervalo interquartil para dados com distribuição não normal. DAP: doença arterial periférica; DCV: doença cardiovascular; DM2: diabetes mellitus tipo 2; IDF: Federação Internacional de Diabetes; IMC: índice de massa corporal; RCV: risco cardiovascular; SM: síndrome metabólica.

A Tabela 2 apresenta os dados do exame físico, das medidas antropométricas e dos exames laboratoriais. A mediana da circunferência abdominal (CA) foi de 105 cm (IIQ: 94–114), e da circunferência do quadril (CQ), de 106 cm (IIQ: 97–114). O IMC mediano foi de 31,62 kg/m^2^. Participantes com fibrose hepática apresentaram CA mais elevada, bem como níveis aumentados de gama-glutamiltransferase (GGT), alanina aminotransferase (ALT) e aspartato aminotransferase (AST).

**Tabela 2:** Exame físico, dados antropométricos e laboratoriais

Variável	População geral (n=114)	Sem fibrose (n=82)	Com fibrose (n=31)	Valor p	DM2 sem fibrose (n=58)	DM2 com fibrose (n=27)	Valor p
**PAS (mmHg)**	130 (128-150)	130 (130-141)	130 (121-155)	0,8729	130 (130-144)	130 (123-160)	0,9133
**PAD (mmHg)**	80 (76-90)	80 (76 - 90)	80 (79 - 90)	0,7163	80 (73-90)	80 (79-90)	0,2349
**CP (cm)**	37 (35-41)	36 (34-41)	40 (35-42)	0,1187	37 (34-42)	40 (35-42)	0,1771
**RCEst (cm)**	0,67 (0,59-0,73)	0,65 (0,59-0,73)	0,67 (0,64-0,73)	0,1579	0,64 (0,58-0,72)	0,67 (0,64-0,73)	0,0897
**CA (cm)**	105 (94-114)	103,50 ± 10,02	111,40 ± 10,02	0,0477	102,20 ± 10,02	107,50 ± 10,02	0,0230
**Quadril (cm)**	106 (97-114)	106 (96-114)	110 (105-120)	0,0682	101 (95-112)	109 (104-118)	0,0352
**IMC (kg/m** ^2^)	31,62 (28,16-36,06)	10,96 ± 5,01	11,88 ± 5,01	0,3284	31,03 ± 5,61	33,23 ± 5,61	0,112
**ALB (g/dl)**	4,4 (4,2-4,7)	4,44 ± 0,27	4,36 ± 0,27	0,3374	4,45 ± 0,27	4,35 ± 0,27	0,1619
**GGT (u/l)**	33 (25-56)	32 (24-44)	54 (29-78)	0,0035	29 (24-44)	54 (30-76)	0,0048
**ALT (u/l)**	22 (16-30)	20 (15-26)	29 (19-46)	0,0025	20 (15-26)	29 (18-44)	0,0082
**AST (u/l)**	22 (18-30)	20 (17-27)	31 (22-41)	0,0002	19 (16-25)	28 (20-38)	0,0012
**BT (mg/dl)**	0,39 (0,28-0,53)	0,37 (0,27-0,53)	0,43 (0,30-0,53)	0,5560	0,34 (0,27-0,53)	0,40 (0,30-0,50)	0,6894
**TFGe (ml/min/1.73m** ^2^)	86,80 (73,62-97,12)	85,22 ± 21,99	86,48 ± 21,99	0,6640	84,77 ± 21,99	82,58 ± 21,99	0,6294
**PCR (mg/dl)**	0,5 (0,3-0,9)	0,43 (0,2-0,7)	0,57 (0,4-1,2)	0,0927	0,4 (0,3-0,9)	0,6 (0,4-1,1)	0,2371
**TSH (mU/l)**	1,8 (1,2-3,5)	1,7 (1,1-3,2)	2,3 (1,4-3,8)	0,1774	1,6 (1,1-3,2)	2,1 (1,3-3,4)	0,4653
**HbA1c (%)**	6,9 (6,0-8,3)	6,6 (5,9-8,2)	7,1 (6,5-8,6)	0,0531	7,5 (6,4-8,6)	7,3 (6,7-9,0)	0,7742
**CT (mg/dl)**	168 (143-191)	169 (141-192)	166 (147-184)	0,9939	165 (139-192)	166 (147-193)	0,7159
**LDL-c (mg/dl)**	95 (75-123)	96 (73-126)	94 (77-112)	0,6677	86 (72-126)	94 (75-112)	0,9847
**HDL-c (mg/dl)**	44 (38-53)	44 (38-53)	45 (38-51)	0,9222	86 (72-126)	45 (38-51)	0,8032
**TG (mg/dl)**	131 (92-179)	135 (92-179)	129 (99-175)	0,9948	129 (93-178)	145 (103-185)	0,4995

Os valores são expressos como média e desvio padrão para dados com distribuição normal e como mediana e intervalo interquartil para dados com distribuição não normal. AC: circunferência abdominal; ALB: albumina; ALT: alanina aminotransferase; AST: aspartato aminotransferase; BT: bilirrubina total; CA: circunferência abdominal; CP: circunferência do pescoço; CT: colesterol total; GGT: gama-glutamiltransferase; HbA1c: hemoglobina glicada; HDL-c: colesterol de lipoproteína de alta densidade; IMC: índice de massa corporal; LDL-c: colesterol de lipoproteína de baixa densidade; PAD: pressão arterial diastólica; PAS: pressão arterial sistólica; PCR: proteína C reativa; RCEst: razão cintura-estatura; TFGe: taxa de filtração glomerular estimada; TG: triglicerídeos; TSH: hormônio estimulador da tireoide.

A esteatose hepática foi observada em 99 participantes (86,8%), e a fibrose hepática esteve presente em 31 participantes (27,2%). Em um participante com DM2, não foi possível realizar a elastografia hepática devido ao biotipo corporal. Embora os valores medianos do CAP tenham sido mais elevados no grupo com fibrose (322 dB/m vs. 292 dB/m), a diferença na gravidade da esteatose entre os grupos não foi estatisticamente significativa. No subgrupo com DM2, a diferença nos valores de CAP entre os grupos foi ainda maior (325 dB/m vs. 290 dB/m), mas também sem significância estatística ( Tabela 3 ).

**Tabela 3:** Exames de imagem hepática

Variável	População geral (n=114)	Sem fibrose (n=82)	Com fibrose (n=31)	Valor p	DM2 sem fibrose (n=58)	DM2 com fibrose (n=27)	Valor p
**Esteatose (n, %)**	99 (86,8)	70 (85,4)	28 (90,3)	0,7023	49 (84,5)	24 (88,9)	0,1052
**Grau de esteatose (n, %)**							
Ausente	15 (13,2)	12 (14,6)	3 (9,7)		9 (15,5)	3 (11,1)	
Leve	22 (19,3)	18 (22)	3 (9,7)	0,2612	13 (22,4)	2 (7,4)	0,1665
Moderada	33 (28,9)	24 (29,3)	9 (29,0)		17 (29,3)	7 (25,9)	
Acentuada	44 (38,6)	28 (34,1)	16 (51,6)		19 (32,8)	15 55,6)	
**Esteatose hepática (FLI** ≥ **60) (n, %)**	59 (51,8)	38 (46,3)	21 (67,7)	0,1881	27 (46,6)	18 (31,0)	0,0651
**CAP**	300 (259,8-342)	292 (259-341)	322 (267-342)	0,5775	290 (259-347)	325 (271-342)	0,3488

Os valores são expressos como média e desvio padrão para dados com distribuição normal, e como mediana e intervalo interquartil para dados com distribuição não normal. CAP: parâmetro de atenuação controlada; FLI: índice de gordura no fígado; DM2: diabetes mellitus tipo 2.

A ultrassonografia carotídea revelou uma mediana de IV de 65 anos (IIQ: 53–79), e placas ateroscleróticas foram detectadas em 33 participantes (28,9%). Não houve diferença significativa na frequência de placas entre os participantes com e sem fibrose. Embora o grupo com fibrose tenha apresentado média total da EMIC (MT EMIC) mais elevada, essa diferença não foi estatisticamente significativa. No entanto, a IV em relação à idade cronológica foi maior no grupo com fibrose, com uma diferença de 8 anos em comparação ao grupo sem fibrose ( Tabela 4 ).

**Tabela 4:** Exame de imagem de carótidas

Variável	População geral (n=114)	Sem fibrose (n=82)	Com fibrose (n=31)	Valor p	DM2 sem fibrose (n=58)	DM2 com fibrose (n=27)	Valor p
MT EMIC (mm)	0,665 (0,580-0,770)	0,66 ± 0,15	0,72 ± 0,15	0,0508	0,653 (0,565-0,768)	0,742 (0,646-0,819)	0,0357
MR EMIC (mm)	0,660 (0,570-0,790)	0,645 (0,550-0,765)	0,700 (0,620-0,800)	0,0948	0,655 (0,550-0,770)	0,740 (0,650-0,805)	0,0463
ML EMIC (mm)	0,665 (0,580-0,770)	0,650 (0,570-0,758)	0,710 (0,590-0,840)	0,0610	0,670 (0,570-0,768)	0,730 (0,630-0,855)	0,0456
Placa (n, %)	33 (28,9%)	22 (26,8%)	10 (32,3%)	0,7357	17 (29,3%)	10 (37,0%)	0,6173
EMIC > p75 (n, %)	42 (36,8%)	29 (35,4%)	13 (41,9%)	0,0508	15 (25,9%)	12 (44,4%)	0,4834
IV (anos)	65 (53–79)	67,18 ± 17,52	73,80 ± 17,52	0,1365	69,59 ± 17,52	73,08 ± 17,52	0,0240

Os valores são expressos como média e desvio padrão para dados com distribuição normal, e como mediana e intervalo interquartil para dados com distribuição não normal. EMIC: espessura médio-intimal da carótida; ML EMIC: EMIC média da carótida comum esquerda; MR EMIC: EMIC média da carótida comum direita; MT EMIC: EMIC média total da carótida comum; DM2: diabetes mellitus tipo 2; IV: idade vascular.

Foi realizada uma subanálise com 85 participantes com DM2, com idade mediana de 64 anos, dos quais 70 (82,3%) eram mulheres. Participantes com DM2 e fibrose hepática apresentaram níveis mais elevados de GGT e transaminases, bem como maior circunferência do quadril ( Tabela 2 ). A esteatose hepática esteve presente em 73 participantes (85,9%) e a fibrose hepática em 27 (31,8%) ( Tabela 3 ).

Nesta subanálise, os participantes com DM2 e fibrose apresentaram IV e MT EMIC mais elevadas em comparação àqueles sem fibrose ( Tabela 4 ). A IV em relação à idade cronológica foi 9 anos maior no grupo com fibrose. Não houve diferença significativa na frequência de placas ateroscleróticas entre os grupos ( Tabela 4 ).

Não foram encontradas correlações significativas entre a MT EMIC e FLI, CAP, GGT, CA, CP ou RCEst. No subgrupo com DM2, observou-se uma correlação negativa fraca, porém estatisticamente significativa, entre a MT EMIC e o FLI. Nenhuma outra correlação significativa foi identificada. Os resultados detalhados estão apresentados na Tabela 5 .

**Tabela 5:** Correlação entre MT-EMIC e variáveis metabólicas na população geral e no grupo com DM2

Variável	População geral (n = 114)	Valor p	DM2 (n = 85)	Valor p
	r		r	
FLI	–0,1483	0,1629	–0,2547	0,0375
CAP	–0,1009	0,2986	–0,1243	0,2658
GGT	0,0015	0,9875	0,1021	0,3740
CA	–0,0462	0,6350	–0,0444	0,6956
CP	–0,0657	0,4970	–0,0229	0,8389
RCEst	0,1200	0,2138	0,0563	0,6177

CA: circunferência abdominal; CAP: parâmetro de atenuação controlada; CP: circunferência do pescoço; DM2: diabetes mellitus tipo 2; EMIC: espessura médio-intimal da carótida; FLI: índice de fígado gorduroso; GGT: gama-glutamiltransferase; RCEst: razão cintura-estatura; r: coeficiente de correlação de Pearson; p: nível de significância (valores p < 0,05 foram considerados estatisticamente significativos).

## Discussão

A DCV acomete quase metade dos indivíduos com DHEADM, sendo a aterosclerose carotídea relatada em aproximadamente 35% dos participantes com doença hepática esteatótica.^24^

Neste estudo prospectivo, avaliou-se a relação entre DHEADM e aterosclerose subclínica utilizando a EMIC, a IV e a presença de placas ateroscleróticas carotídeas como marcadores preditivos. Esses dados ainda são escassos na literatura brasileira. Ademais, até onde se tem conhecimento, este é o primeiro estudo realizado em uma população brasileira com DHEADM a avaliar a IV com base na EMIC.

Entre os 114 participantes com risco para DHEADM, recrutados em um ambulatório de endocrinologia, 86,8% foram diagnosticados com esteatose hepática e 27,2% com fibrose hepática. Na população geral, a prevalência de esteatose varia entre 30% e 40%, enquanto a fibrose hepática acomete aproximadamente 5% a 10%.^2^ A maior prevalência observada no presente estudo pode ser explicada pelas características da amostra, que incluiu elevada proporção de participantes com DM2 (75,4%), fator de risco reconhecido tanto para esteatose quanto para fibrose.^1^ Além disso, uma parcela substancial da população estudada era composta por mulheres pós-menopáusicas, grupo no qual a relação entre fibrose hepática e DCV permanece pouco explorada.^25^

A gravidade da DHEADM está associada ao aumento do risco de aterosclerose e mortalidade,^12^ sendo os pacientes com DM2 um subgrupo particularmente vulnerável.^5^ No entanto, diversos fatores de risco são compartilhados entre a DHEADM e a aterosclerose — como obesidade e SM —, podendo atuar como fatores de confusão. Neste estudo, não foram observadas diferenças significativas nesses potenciais fatores de confusão ao comparar participantes com e sem fibrose hepática, inclusive na subanálise do subgrupo com DM2. Os grupos foram semelhantes quanto à prevalência de obesidade e a outras medidas antropométricas de adiposidade, com exceção da circunferência do quadril, que foi maior em participantes diabéticos com fibrose. Não foram encontradas diferenças em relação à idade, sexo, tabagismo, parâmetros laboratoriais ou uso de estatinas.

A gama-glutamiltransferase (GGT) é uma enzima envolvida no sistema da glutationa, o qual atua como um dos principais mecanismos antioxidantes do organismo. Níveis elevados de GGT em indivíduos com fibrose podem indicar a presença de estresse oxidativo. De forma semelhante, o risco cardiovascular está relacionado ao estresse oxidativo e à inflamação crônica — ambos refletidos pelo aumento dos níveis de GGT. Assim, a GGT pode representar um biomarcador disponível, de baixo custo, e elevado tanto na fibrose hepática quanto na DCV.^26 , 27^

O presente estudo não demonstrou associação significativa entre fibrose hepática e a presença de placas ateroscleróticas detectadas por ultrassonografia carotídea, achado consistente com resultados de outros estudos.^28 - 30^ Por outro lado, alguns estudos prévios relataram tal associação. Essas discrepâncias podem ser explicadas por diferenças metodológicas, bem como por características clínicas, étnicas e demográficas das populações avaliadas, além do tamanho amostral reduzido.^31 - 33^

Em nossa amostra, a maioria dos participantes era composta por mulheres, muitas das quais faziam uso de estatinas e apresentavam níveis de hemoglobina glicada próximos aos valores-alvo. Esses fatores foram semelhantes entre os grupos com e sem fibrose. Além disso, os dados referentes à população brasileira ainda são escassos, uma vez que a maioria dos estudos se concentra em populações asiáticas.^2^

A elevação da EMIC é um fator de risco independente para eventos cardiovasculares e cerebrovasculares.^8^ Em nossa população, a EMIC foi avaliada e observou-se que foi mais elevada nos participantes com fibrose em comparação àqueles sem fibrose, especialmente no subgrupo com DM2.

Alguns autores não identificaram associação entre DHEADM e aumento da EMIC, inclusive em estudos realizados com participantes com DM2.^30 , 33 , 34^ Por outro lado, outros estudos demonstraram uma associação positiva entre DHEADM e EMIC aumentada.^31 , 32 , 35^

A ultrassonografia de carótidas pode ser utilizada para avaliar a IV por meio de parâmetros como EMIC, distensibilidade carotídea e velocidade da onda de pulso (VOP). Cada uma dessas medidas fornece informações relevantes sobre as condições funcionais e estruturais das artérias, contribuindo para uma avaliação mais abrangente do envelhecimento vascular. Neste estudo, a IV foi superior à idade cronológica em participantes com fibrose hepática, tanto na amostra total quanto no subgrupo com DM2 — com diferença estatisticamente significativa neste último ( Tabela 4 ). Os participantes com DM2 e fibrose apresentaram IV nove anos maior do que a IC. Esse achado sugere uma possível associação entre fibrose hepática e envelhecimento cardiovascular acelerado em indivíduos com DM2. Um estudo longitudinal brasileiro conduzido com 291 pacientes com DM2 e DHEADM utilizou a VOP carotídeo-femoral para avaliar a IV. O estudo demonstrou que o aumento da rigidez aórtica, ou sua progressão ao longo do tempo, foi preditor do desenvolvimento de fibrose hepática avançada.^36^ Além disso, outro estudo brasileiro evidenciou IV elevada em indivíduos com diabetes tipo 1 (DM1).^37^ Tomados em conjunto, esses achados sugerem que o diabetes mellitus contribui para o aumento da IV, refletindo maior risco cardiovascular e disfunção endotelial.^37^

Zhou et al. (2018) demonstraram que a DHEADM está associada de forma independente a uma maior prevalência de DCV em pacientes com DM2, e propuseram a DHEADM como fator de risco adicional para DCV nessa população.^38^

Uma metanálise recente com 7.951 participantes sob risco para DHEADM demonstrou que a prevalência de aterosclerose carotídea variou de acordo com a região geográfica, sendo mais alta na Europa (45%), seguida pela América do Norte (41%), Ásia (36%) e, por fim, pelo Oriente Médio (19%).^24^ Esses achados ressaltam a escassez de estudos com foco na população da América Latina.

Sinn et al. (2016) observaram que, entre indivíduos com DHEADM, a frequência de placas carotídeas foi maior naqueles com menor média de idade.^39^ A associação entre DHEADM e presença de placas carotídeas foi mais acentuada em adultos jovens do que em idosos. No entanto, os autores destacaram que a sensibilidade e a especificidade da DHEADM para predizer placas carotídeas são limitadas. Apesar disso, não recomendaram o rastreamento rotineiro da DHEADM na população geral com o único propósito de avaliação do risco cardiovascular.^39^ Shao et al. (2020) propuseram que a idade e o IMC podem atuar como preditores de risco cardiovascular em indivíduos com DHEADM.^40^

Embora a quantificação da esteatose pelo CAP não tenha apresentado diferença estatisticamente significativa entre os participantes com e sem fibrose — inclusive no subgrupo com DM2 —, os valores mais elevados de CAP foram observados nos indivíduos com fibrose, o que sugere uma doença mais avançada. Eddowes et al. (2019) demonstraram que, embora o CAP tenha poder discriminatório limitado para diferenciar esteatose grau 2 de grau 3 (moderada vs. grave), apresenta boa acurácia para o diagnóstico de esteatose quando comparado à biópsia hepática.^21^ Portanto, a quantificação da esteatose isoladamente não é considerada um indicador confiável da gravidade da doença. Ainda assim, nossos achados podem indicar que, em uma população sob risco para DHEADM, valores elevados de CAP possam estar associados a formas mais graves da doença esteatótica.

Entre as limitações deste estudo, destaca-se o delineamento transversal, que impede o estabelecimento de relações causais entre DHEADM e aumento da EMIC. A especificidade regional da amostra também limita a generalização dos achados para a população brasileira e global. Ademais, o tamanho amostral relativamente pequeno e a amostragem por conveniência em um hospital de atenção terciária podem introduzir viés de seleção, com possível super-representação de casos mais graves e limitação na aplicabilidade dos resultados à população geral. Por outro lado, o estudo apresenta delineamento prospectivo, e todos os exames foram realizados por um único examinador experiente. Apesar dessas limitações, o estudo possui pontos fortes, como a padronização metodológica e a realização de todas as avaliações de imagem por um único examinador experiente, o que assegura maior consistência e confiabilidade dos dados.

O diagnóstico da doença hepática e de sua progressão (esteatose e fibrose), neste estudo, foi baseado em ultrassonografia hepática e elastografia, e não na biópsia hepática — atual padrão-ouro. No entanto, o uso de um procedimento invasivo como a biópsia em larga escala é inviável e representa risco inaceitável aos pacientes.

Neste estudo, conduzido com população brasileira da região metropolitana de Niterói, os participantes com fibrose hepática moderada a avançada associada à DHEADM apresentaram EMIC e IV mais elevadas, com significância estatística observada no subgrupo com DM2.

Apesar das discussões ainda em curso na literatura internacional, a DHEADM continua sendo um fator de risco subestimado e independente para DCV aterosclerótica. Compartilha diversos mecanismos fisiopatológicos com a DCV, incluindo inflamação sistêmica de baixo grau, disfunção endotelial e resistência à insulina.^41 - 44^

Dadas as variações regionais nas características populacionais, são necessários novos estudos para avaliar com maior precisão a associação entre fibrose hepática decorrente da DHEADM e aumento da EMIC, da IV e da presença de placas ateroscleróticas.

No contexto da avaliação global de risco cardiovascular em pacientes com DM2, a fibrose hepática associada à DHEADM pode representar um fator relevante a ser considerado. Estudos adicionais são necessários para confirmar essa hipótese.

## Conclusões

Este estudo destaca o potencial papel da fibrose hepática decorrente da DHEADM — especialmente em indivíduos com DM2 — como um marcador intermediário de risco cardiovascular. Entre os participantes brasileiros sob risco para DHEADM, aqueles com DM2 e fibrose hepática apresentaram EMIC e IV mais elevadas, sugerindo envelhecimento vascular precoce e risco cardiovascular aumentado. Esses achados reforçam a importância da incorporação de indicadores intermediários, como a EMIC e a IV, em conjunto com os escores tradicionais de risco cardiovascular, na avaliação abrangente de indivíduos com DHEADM.

Disponibilidade de Dados

Todo o conjunto de dados que dá suporte aos resultados deste estudo está disponível mediante solicitação ao autor correspondente. O conjunto de dados não está publicamente disponível, pois de acordo com os padrões éticos brasileiros para pesquisa em seres humanos, não podemos disponibilizar publicamente o conjunto completo de dados clínicos devido ao compromisso de proteger a privacidade e a confidencialidade dos participantes. Essa abordagem segue a Resolução 466/2012 do Conselho Nacional de Saúde e a Lei Geral de Proteção de Dados (LGPD - Lei 13.709/2018). No entanto, acreditamos firmemente na transparência científica e estamos comprometidos com o compartilhamento responsável de dados. Os conjuntos de dados pseudonimizados que fundamentam os resultados apresentados neste artigo estarão disponíveis mediante solicitação razoável ao(s) autor(es) correspondente(s), sujeitos à aprovação do Comitê de Ética em Pesquisa original que autorizou nosso estudo.
