# Supplementary material for: Relação entre Fibrose Hepática Decorrente da Doença Hepática Gordurosa Associada à Disfunção Metabólica e Aterosclerose Subclínica
Source: Arq Bras Cardiol. 2025 Aug 20;122(8):e20240856. [Article in Portuguese] doi: 10.36660/abc.20240856 (PMC12671716; doi:10.36660/abc.20240856)
Supplement: Translation

# Translation: Relationship between Liver Fibrosis Due to Metabolic Dysfunction-Associated Steatotic Liver Disease and Subclinical Atherosclerosis

**DOI:** 10.36660/abc.20240856i

**Authors:** Daniele Araujo de Azeredo Coutinho, Jenaine Rosa Godinho, Raphael Carreiro Moura, Rogério Martins Oliveira, Hevila Faria Passos, Jordana de Paula Felipe Mendes, Carlos Roberto Moraes de Andrade, Luis Guillermo Coca-Velarde, Maria Auxiliadora Nogueira Saad, Priscila Pollo Flores, Debora Vieira Soares

**Affiliations:** 1 Niterói RJ Brazil Universidade Federal Fluminense, Niterói, RJ – Brazil; 2 Niterói RJ Brazil Universidade Federal Fluminense - Hospital Universitário Antônio Pedro, Niterói, RJ – Brazil

**Keywords:** Atherosclerosis, Type 2 Diabetes Mellitus, Liver Cirrhosis, Heart Disease Risk Factors

## Abstract

**Background:**

Metabolic dysfunction-associated steatotic liver disease (MASLD) is the most prevalent liver disease worldwide. MASLD can progress to fibrosis and related complications, with cardiovascular disease being the leading cause of death in affected individuals. Carotid atherosclerosis markers can predict cardiovascular outcomes, underscoring the relevance of their association with liver fibrosis.

**Objective:**

To evaluate the relationship between liver fibrosis in MASLD and subclinical atherosclerosis by assessing carotid intima-media thickness (cIMT), vascular age (VA), and the presence of atherosclerotic plaques.

**Methods:**

This prospective study included participants at risk for MASLD. Liver steatosis and fibrosis were assessed via ultrasound and liver elastography using controlled attenuation parameter measurements. Carotid atherosclerosis was evaluated through carotid ultrasound, cIMT measurements, and VA. A significance level of 5% (p < 0.05) was adopted.

**Results:**

A total of 114 participants were included, with a median age of 64 years (IQR: 55–68), and 96 (84%) were women. Steatosis was identified in 99 participants (86.8%) and fibrosis in 31 (27.2%). Atherosclerotic plaques were present in 33 participants (28.9%), with no significant difference in frequency between groups. However, the fibrosis group showed higher cIMT and elevated VA. In a subgroup analysis of 85 participants with type 2 diabetes mellitus (T2DM), 27 (31.8%) had fibrosis. These individuals had a higher cIMT (0.742 mm vs. 0.653 mm; p < 0.05), and VA exceeded chronological age by 9 years in the fibrosis group compared to those without fibrosis (p < 0.05).

**Conclusion:**

This study demonstrates that individuals with MASLD and liver fibrosis exhibit greater vascular aging and increased cardiovascular risk, as reflected by elevated cIMT and VA.

## Introduction

Metabolic dysfunction-associated steatotic liver disease (MASLD)^1^is a highly prevalent condition, affecting approximately 30% of the global population and up to 44% in Latin America.^2^MASLD encompasses a spectrum ranging from simple steatosis to steatohepatitis (MASH), fibrosis, and ultimately liver cirrhosis. In the general population, about 10% of individuals with MASLD progress to MASH, fibrosis, and advanced liver disease. However, this proportion is significantly higher among those with type 2 diabetes mellitus (T2DM) and obesity.^1 , 3 , 4^

Overall mortality is elevated in individuals with MASLD and fibrosis, with cardiovascular disease (CVD) being the leading cause of death.^3 , 5^In cases of advanced fibrosis and cirrhosis, liver-related mortality becomes more prominent.^3^

The main metabolic risk factors for MASLD are associated with insulin resistance, metabolic syndrome (MetS), visceral obesity, diabetes mellitus, and atherogenic dyslipidemia.^1 , 3^While some of the cardiovascular risk can be attributed to these comorbidities, the diagnosis of MASLD with fibrosis is independently associated with increased cardiovascular mortality.^6 , 7^

Previous studies have shown that measuring carotid intima-media thickness (cIMT) by carotid ultrasound is an independent predictor of cardiovascular risk. cIMT is associated with the likelihood of future coronary and cerebrovascular ischemic events, even in asymptomatic individuals.^8^Conversely, MASLD and the presence of liver fibrosis may also serve as potential predictors of subclinical atherosclerosis.^9^Several studies have reported an association between subclinical CVD and liver fibrosis resulting from MASLD.^9 , 10^

The aim of the present study was to evaluate the relationship between liver fibrosis in MASLD and subclinical atherosclerosis by assessing cIMT, calculating vascular age (VA), and determining the frequency of atherosclerotic plaques.

## Methods

This was a prospective, observational study with cross-sectional data analysis. Participants were recruited from the Endocrinology outpatient clinic of a university hospital between March 2020 and August 2021. Recruitment was interrupted due to the COVID-19 pandemic. The study was approved by the local Research Ethics Committee under protocol number CAAE: 51731721.7.0000.5243. All participants provided written informed consent.

Inclusion criteria were age over 18 years and the presence of risk factors for MASLD, including prediabetes, T2DM, obesity, and MetS. MetS was defined according to the criteria of the International Diabetes Federation (IDF), which require central obesity (waist circumference > 90 cm in men and > 80 cm in women) plus two or more of the following: triglycerides ≥ 150 mg/dL or use of lipid-lowering agents; HDL cholesterol < 40 mg/dL in men and < 50 mg/dL in women or use of lipid-lowering agents; systolic blood pressure ≥ 130 mmHg and/or diastolic blood pressure ≥ 85 mmHg or use of antihypertensive medications; fasting blood glucose ≥ 100 mg/dL or a previous diagnosis of T2DM.^10^

Exclusion criteria included a current or past history of alcohol consumption > 14 g/day for women and > 20 g/day for men, cancer, use of systemic corticosteroids or androgens, menopausal hormone therapy, history of bariatric surgery, pregnancy, and chronic kidney disease (CKD) with a glomerular filtration rate (GFR) < 60 mL/min/1.73 m^2^, as calculated using the Chronic Kidney Disease Epidemiology Collaboration (CKD-EPI) equation.^11^

Regarding liver diseases, individuals with viral hepatitis (e.g., hepatitis B surface antigen, hepatitis B surface antibody, hepatitis B core antibody, and hepatitis C virus antibody) and autoimmune hepatitis were excluded. Autoimmune hepatitis was screened by measuring IgG and antinuclear antibodies. In patients with elevated liver enzymes and at least one positive or abnormal result, additional tests were performed, including anti-smooth muscle antibody and liver-kidney microsomal type 1 antibody measurements. Individuals with hepatocellular carcinoma, Child-Pugh class C cirrhosis, or decompensated cirrhosis were also excluded.

Participants were categorized into groups with and without liver fibrosis. The presence of liver fibrosis was defined as liver stiffness ≥ 7.1 kPa, which corresponds to stage F2 fibrosis on liver biopsy, according to the Metavir scoring system.^12 , 13^A subgroup analysis was also performed for participants with T2DM.

Demographic characteristics, lifestyle factors, medication use, and prior history of CVD — including stroke, coronary artery disease, and peripheral arterial disease (PAD) — were recorded from medical charts.

Clinical assessments included body weight measurement using a calibrated Filizola^®^anthropometric scale (São Paulo, SP, Brazil), certified by Instituto Nacional de Metrologia, Normalização e Qualidade Industrial (Inmetro). Height was measured with a stadiometer, with participants standing upright. Waist circumference (WC) was measured using a nonelastic SANNY measuring tape (São Bernardo do Campo, SP, Brazil) at the midpoint between the iliac crest and the lower rib, with the participant standing and at the end of a normal exhalation.^14^Neck circumference (NC) was measured at the base of the neck, just below the laryngeal prominence, with the participant standing, looking straight ahead, and shoulders relaxed. NC was considered high if ≥ 35.5 cm for men and ≥ 32 cm for women.^15^The waist-to-height ratio (WHtR) was considered elevated if ≥ 0.5. Body mass index (BMI) was calculated, and obesity was defined as BMI ≥ 30 kg/m^2^, according to World Health Organization (WHO) criteria.^14^

Blood samples were collected after a 12-hour overnight fast. The following laboratory tests were performed using the immunoturbidimetric method: fasting glucose, total cholesterol (TC), high-density lipoprotein cholesterol (HDL-c), triglycerides (TG), liver enzymes, urea, creatinine, bilirubin, ultrasensitive C-reactive protein, and uric acid. Ferritin, thyroid-stimulating hormone (TSH), and free T4 were measured using a chemiluminescence immunoassay with commercial kits from the Alinity S System (São Paulo, Brazil). Low-density lipoprotein cholesterol (LDL-c) was estimated using the Friedewald equation. Platelet counts were determined using the Unicel^®^DxH 800, Coulter^®^Cellular Analysis System.

Liver steatosis was assessed using liver ultrasound and transient elastography, including controlled attenuation parameter (CAP) measurements with the FibroScan^®^Touch 502 model. All exams were performed after a 4-hour fast by the same experienced operator. Steatosis was classified as mild, moderate, or severe according to the Saadeh classification.^16^The Fatty Liver Index (FLI-score) was also used to estimate the probability of steatohepatitis, with an FLI-score ≥ 4 points indicating a high probability.^17 , 18^

Steatosis quantification by CAP was categorized as follows: <236 dB/m indicated absent steatosis or <5% liver fat; 236–279 dB/m indicated mild steatosis (5–33%); 279–302 dB/m indicated moderate steatosis (33–66%); and ≥302 dB/m indicated marked steatosis (>66%).^19^

Liver fibrosis was assessed through liver stiffness measurements using one-dimensional transient elastography (FibroScan^®^, Echosens, France) with M or XL probes, selected based on the participant's body type, and by Acoustic Radiation Force Impulse two-dimensional shear wave elastography (Canon^®^Aplio i800, Japan). Examinations were performed after a 4-hour fast by the same experienced operator. Fibrosis staging was inferred using the METAVIR semi-quantitative score, based on the following liver stiffness values: F0 or F1: < 7.1 kPa; F2: ≥ 7.1 kPa and ≤ 9.5 kPa; F3: > 9.5 kPa and ≤ 12.5 kPa; F4: > 12.5 kPa.^20 , 21^

Carotid ultrasound evaluations were performed by the same experienced examiner, who was blinded to the participant's group assignment. The cIMT was measured digitally using a commercially available EPIQ7 ultrasound system (Philips^®^), equipped with an L12–13 MHz linear transducer. Using Qlab software (Philips^®^), cIMT was measured in millimeters, with precision to three decimal places. Measurements followed the standardized technique outlined by the Department of Cardiovascular Imaging of Brazilian Society of Cardiology.^22^

A transverse scan of the carotid arteries and their extracranial branches was conducted bilaterally to identify visible plaques. Subsequently, with the transducer placed longitudinally along the carotid artery and using the semi-automatic IMT detection program, cIMT measurements were obtained from the distal wall of the common carotid artery, within the last 10 mm proximal to the carotid bulb, at three different angles: posterior, lateral, and anterior. This resulted in six measurements — three on the right and three on the left. Mean values were calculated for the left (ML cIMT), right (MR cIMT), and combined sides (MT cIMT).

An increased IMT was defined as a value above the 75th percentile on at least one side. Reference values were based on the vascular ultrasound guidelines of the Department of Cardiovascular Imaging of the Brazilian Society of Cardiology.^22^Different tables were applied based on the participant's age: for those under 40 years, the CAPS table was used, which does not differentiate by ethnicity; for participants aged 40 to 65, the ELSA-Brazil table was applied, with reference values for individuals of brown ethnicity, reflecting the study population; for those over 65 years, the MESA table for the Hispanic population was used.^22^

To calculate VA, the average cIMT from each side was used. The participant's VA was defined as the age at which their cIMT value corresponded to the 50th percentile bilaterally. In cases where the VA differed between the right and left sides, the higher value was considered.^22^

Cardiovascular risk stratification was performed using the risk calculator proposed by the Brazilian Society of Cardiology, which classifies cardiovascular risk into four categories: low, moderate, high, and very high.^23^

### Statistical analysis

Results are presented as median and interquartile range (p25–p75) for continuous variables with non-normal distribution, mean ± standard deviation for normally distributed continuous variables, and absolute (n) and relative (%) frequencies for categorical variables. The Shapiro-Wilk test was used to assess the normality of numerical data.

For comparisons between groups, the independent samples t-test was used for normally distributed variables, while the Mann-Whitney *U* test was applied for non-normally distributed data. Categorical variables were analyzed using Fisher's exact test, depending on expected frequencies.

After testing for normality, Spearman's correlation was used to assess the relationship between continuous variables. A p-value < 0.05 was considered statistically significant. All analyses were conducted using R software, version 3.6.1 for Windows.

## Results

The main findings of this study are illustrated in Central Illustration, which visually summarizes the most relevant data presented in this article.

A total of 114 participants were included, of whom 96 (84%) were women. The median age was 64 years. Table 1 presents the baseline characteristics of the study population. No significant differences were observed between the fibrosis and non-fibrosis groups regarding age, sex, ethnicity, or associated comorbidities.

**Table 1:** General data and comorbidities

Variable	General population (n=114)	Without fibrosis (n=82)	With fibrosis (n=31)	p-value	T2DM without fibrosis (n=58)	T2DM with fibrosis (n=27)	p-value
**Age (years)**	64 (55-68)	63 (55-68)	65 (58-69)	0.2197	64 (57-68)	66 (62-70)	0.0829
**Female (n, %)**	96 (84)	67 (81.7)	28 (90.3)	0.4074	45 (77.6)	25 (92.6)	0.1286
**Hypertension (n, %)**	101 (88.6)	70 (85.4)	30 (96.8)	0.1721	51 (87.9)	27 (100)	0.0916
**CVD (n, %)**	14 (12.3)	11 (13.4)	3 (9.7)	0.8274	8 (13.8)	3 (11.1)	0.0777
**PAD (n, %)**	7 (6.1)	3 (3.7)	4 (12.9)	0.8273	2 (3.5)	4 (14.8)	0.1471
**Dyslipidemia (n, %)**	96 (84.2)	70 (85.4)	25 (80.6)	0.7461	52 (89.7)	23 (85.2)	0.7189
**Pre-T2DM (n, %)**	19 (16.7)	17 (20.7)	2 (6.5)	0.1262	-	-	-
**T2DM (n, %)**	86 (75.4)	58 (70.7)	27 (87.1)	0.1203	-	-	-
**Overweight (n, %)**	26 (22.8)	22 (26.8)	4 (12.9)	0.1872	17 (29.3)	3 (11.1)	0.0985
**Obesity (n, %)**	65 (57.0)	45 (54.9)	20 (64.5)	0.1805	27 (46.6)	18 (66.7)	0.1047
**MetS IDF (n, %)**	71 (62.3)	50 (61)	20 (64.5)	0.8976	35 (60.3)	19 (70.4)	0.4702
**Use of statin (n, %)**	96 (85)	69 (85.2)	26 (83.9)	1	50 (86.2)	24 (88.9)	1
**Menopause (n, %)**	85 (90.4)	59 (89.4)	25 (92.6)	0.9305	40 (90.9)	23 (95.8)	0.6493
**Smoking (n, %)**							
Never smoked	76 (66.7)	55 (67.1)	21 (67.7)	0.9931	39 (67.2)	17 (63)	0.8234
Current smoker	4 (3.5)	3 (3.7)	1 (3.2)		1 (1.7)	1 (3.7)	
Former smoker	34 (29.8)	24 (29.3)	9 (29.0)		18 (31.0)	9 (33.3)	
**Physical activity (n, %)**							
Sedentary	81 (71.1)	55 (67.1)	25 (80.6)	0.1270	37 (63.8)	23 (85.2)	0.0585
> 150 min/week	22 (19.3)	18 (22)	4 (12.9)		13 (22.4)	2 (7.4)	
< 150 min/week	10 (8.8)	9 (11)	1 (3.2)		8 (13.8)	1 (3.7)	
**Microalbuminuria (n, %)**	9 (7.9)	4 (4.9)	5 (16.1)	0.1137	4 (6.9)	5 (18.5)	0.1351
Retinopathies (n, %)				0.5861			0.9468
CVR (n, %):	4 (3.5)	2 (2.4)	2 (6.5)		0 (0)	0 (0)	
Low	7 (6.1)	4 (4.9)	3 (9.7)		3 (8.6)	1 (3.7)	
Moderate	89 (78.1)	65 (79.3)	23 (74.2)		47 (81.0)	23 (85.2)	
High	12 (10.5)	9 (11)	3 (9.7)		6 (10.3)	3 (11.1)	
Very high							

Values are expressed as mean and standard deviation for normally distributed data, and as median and interquartile range for non-normally distributed data. CVD: cardiovascular disease; CVR: cardiovascular risk; IDF: International Diabetes Federation; MetS: metabolic syndrome; PAD: peripheral arterial disease; T2DM: type 2 diabetes mellitus.

Table 2 presents data from the physical examination, anthropometric measurements, and laboratory tests. The median abdominal circumference (AC) and hip circumference (HC) were 105 cm (IQR: 94–114) and 106 cm (IQR: 97–114), respectively. The median BMI was 31.62 kg/m^2^. Participants with liver fibrosis had higher AC, as well as elevated levels of gamma-glutamyl transferase (GGT), alanine aminotransferase (ALT), and aspartate aminotransferase (AST).

**Table 2:** Physical exam, anthropometric data, and laboratory tests

Variable	General population(n=114)	Without fibrosis (n=82)	With fibrosis (n=31)	p- value	T2DM without fibrosis (n=58)	T2DM with fibrosis (n=27)	p- value
**SBP (mmHg)**	130 (128-150)	130 (130-141)	130 (121-155)	0.8729	130 (130-144)	130 (123-160)	0.9133
**DBP (mmHg)**	80 (76-90)	80 (76 - 90)	80 (79 - 90)	0.7163	80 (73-90)	80 (79-90)	0.2349
**NC (cm)**	37 (35-41)	36 (34-41)	40 (35-42)	0.1187	37 (34-42)	40 (35-42)	0.1771
**WHtR (cm)**	0.67 (0.59-0.73)	0.65 (0.59-0.73)	0.67 (0.64-0.73)	0.1579	0.64 (0.58-0.72)	0.67 (0.64-0.73)	0.0897
**AC (cm)**	105 (94-114)	103.50 ± 10.02	111.40 ± 10.02	0.0477	102.20 ± 10.02	107.50 ± 10.02	0.0230
**Hip (cm)**	106 (97-114)	106 (96-114)	110 (105-120)	0.0682	101 (95-112)	109 (104-118)	0.0352
**BMI (Kg/m** ^2^)	31.62 (28.16-36.06)	10.96 ± 5.01	11.88 ± 5.01	0.3284	31.03 ± 5.61	33.23 ± 5.61	0.112
**ALB (g/dL)**	4.4 (4.2-4.7)	4.44 ± 0.27	4.36 ± 0.27	0.3374	4.45 ± 0.27	4.35 ± 0.27	0.1619
**GGT (u/L)**	33 (25-56)	32 (24-44)	54 (29-78)	0.0035	29 (24-44)	54 (30-76)	0.0048
**ALT (u/L)**	22 (16-30)	20 (15-26)	29 (19-46)	0.0025	20 (15-26)	29 (18-44)	0.0082
**AST (u/L)**	22 (18-30)	20 (17-27)	31 (22-41)	0.0002	19 (16-25)	28 (20-38)	0.0012
**TB (mg/dL)**	0.39 (0.28-0.53)	0.37 (0.27-0.53)	0.43 (0.30-0.53)	0.5560	0.34 (0.27-0.53)	0.40 (0.30-0.50)	0.6894
**eGFR (mL/min/1.73m** ^2^)	86.80 (73.62-97.12)	85.22 ± 21.99	86.48 ± 21.99	0.6640	84.77 ± 21.99	82.58 ± 21.99	0.6294
**CRP (mg/dL)**	0.5 (0.3-0.9)	0.43 (0.2-0.7)	0.57 (0.4-1.2)	0.0927	0.4 (0.3-0.9)	0.6 (0.4-1.1)	0.2371
**TSH (mU/L)**	1.8 (1.2-3.5)	1.7 (1.1-3.2)	2.3 (1.4-3.8)	0.1774	1.6 (1.1-3.2)	2.1 (1.3-3.4)	0.4653
**HbA1c (%)**	6.9 (6.0-8.3)	6.6 (5.9-8.2)	7.1 (6.5-8.6)	0.0531	7.5 (6.4-8.6)	7.3 (6.7-9.0)	0.7742
**TC (mg/dL)**	168 (143-191)	169 (141-192)	166 (147-184)	0.9939	165 (139-192)	166 (147-193)	0.7159
**LDL-c (mg/dL)**	95 (75-123)	96 (73-126)	94 (77-112)	0.6677	86 (72-126)	94 (75-112)	0.9847
**HDL-c (mg/dL)**	44 (38-53)	44 (38-53)	45 (38-51)	0.9222	86 (72-126)	45 (38-51)	0.8032
**TG (mg/dL)**	131 (92-179)	135 (92-179)	129 (99-175)	0.9948	129 (93-178)	145 (103-185)	0.4995

Values are expressed as mean and standard deviation for normally distributed data, and as median and interquartile range for non-normally distributed data. AC: abdominal circumference; ALB: albumin; ALT: alanine aminotransferase; AST: aspartate aminotransferase; BMI: body mass index; CRP: C-reactive protein; DBP: diastolic blood pressure; eGFR: estimated glomerular filtration rate; GGT: gamma-glutamyl transferase; HbA1c: glycated hemoglobin; HDL-c: high-density lipoprotein cholesterol; LDL-c: low-density lipoprotein cholesterol; NC: neck circumference; SBP: systolic blood pressure; TC: total cholesterol; TG: triglycerides; TSH: thyroid-stimulating hormone; TB: total bilirubin; WHtR: waist-to-height ratio.

Liver steatosis was observed in 99 participants (86.8%), and liver fibrosis was present in 31 participants (27.2%). In one participant with T2DM, liver elastography could not be performed due to body habitus. Although the median CAP values were higher in the fibrosis group (322 dB/m vs. 292 dB/m), the difference in steatosis severity between groups was not statistically significant. In the T2DM subgroup, the difference in CAP values between groups was even greater (325 dB/m vs. 290 dB/m), yet remained statistically non-significant ( Table 3 ).

**Table 3:** Liver imaging exams

Variable	General population (n=114)	Without fibrosis (n=82)	With fibrosis (n=31)	p- value	T2DM without fibrosis (n=58)	T2DM with fibrosis (n=27)	p- value
**Steatosis (n, %)**	99 (86.8)	70 (85.4)	28 (90.3)	0.7023	49 (84.5)	24 (88.9)	0.1052
**Steatosis degree (n, %)**							
Absent	15 (13.2)	12 (14.6)	3 (9.7)		9 (15.5)	3 (11.1)	
Light	22 (19.3)	18 (22)	3 (9.7)	0.2612	13 (22.4)	2 (7.4)	0.1665
Moderate	33 (28.9)	24 (29.3)	9 (29.0)		17 (29.3)	7 (25.9)	
High	44 (38.6)	28 (34.1)	16 (51.6)		19 (32.8)	15 55.6)	
**Hepatic steatosis (FLI** ≥ **60) (n, %)**	59 (51.8)	38 (46.3)	21 (67.7)	0.1881	27 (46.6)	18 (31.0)	0.0651
**CAP**	300 (259.8-342)	292 (259-341)	322 (267-342)	0.5775	290 (259-347)	325 (271-342)	0.3488

Values are expressed as mean and standard deviation for normally distributed data, and as median and interquartile range for non-normally distributed data. CAP: controlled attenuation parameter; FLI: fatty liver index; T2DM: type 2 diabetes mellitus.

Carotid ultrasound revealed a median VA of 65 years (IQR: 53–79), and atherosclerotic plaques were detected in 33 participants (28.9%). There was no significant difference in plaque frequency between participants with and without fibrosis. Although the fibrosis group had a higher total mean cIMT (MT cIMT), the difference was not statistically significant. However, VA in relation to chronological age (CA) was higher in the fibrosis group, differing by 8 years compared to the non-fibrosis group ( Table 4 ).

**Table 4:** Carotid imaging exam

Variable	General population (n=114)	Without fibrosis (n=82)	With fibrosis (n=31)	p-value	T2DM without fibrosis (n=58)	T2DM with fibrosis (n=27)	p-value
MT cIMT (mm)	0.665 (0.580-0.770)	0.66 ± 0.15	0.72 ± 0.15	0.0508	0.653 (0.565-0.768)	0.742 (0.646-0.819)	0.0357
MR cIMT (mm)	0.660 (0.570-0.790)	0.645 (0.550-0.765)	0.700 (0.620-0.800)	0.0948	0.655 (0.550-0.770)	0.740 (0.650-0.805)	0.0463
ML cIMT (mm)	0.665 (0.580-0.770)	0.650 (0.570-0.758)	0.710 0.590-0.840)	0.0610	0.670 (0.570-0.768)	0.730 (0.630-0.855)	0.0456
Plaque (n, %)	33 (28.9%)	22 (26.8%)	10 (32.3%)	0.7357	17 (29.3%)	10 (37.0%)	0.6173
cIMT > p75 (n, %)	42 (36.8%)	29 (35.4%)	13 (41.9%)	0.0508	15 (25.9%)	12 (44.4%)	0.4834
VA (years)	65 (53–79)	67.18 ± 17.52	73.80 ± 17.52	0.1365	69.59 ± 17.52	73.08 ± 17.52	0.0240

Values are expressed as mean and standard deviation for normally distributed data, and as median and interquartile range for non-normally distributed data. cIMT: carotid intima-media thickness; ML cIMT: mean left common carotid artery intima-media thickness; MR cIMT: mean right common carotid artery intima-media thickness; MT cIMT: total mean common carotid artery intima-media thickness; T2DM: type 2 diabetes mellitus; VA: vascular age.

A sub-analysis was conducted on 85 participants with T2DM, with a median age of 64 years, of whom 70 (82.3%) were women. Participants with T2DM and liver fibrosis had higher levels of GGT and transaminases as well as greater HC ( Table 2 ). Liver steatosis was present in 73 participants (85.9%), and liver fibrosis in 27 (31.8%) ( Table 3 ).

In this sub-analysis, participants with T2DM and fibrosis had higher VA and higher total mean cIMT (MT cIMT) compared to those without fibrosis ( Table 4 ). VA in relation to CA was 9 years higher in the fibrosis group. There was no significant difference in the frequency of atherosclerotic plaques between the groups ( Table 4 ).

No significant correlation was found between MT cIMT and FLI, CAP, GGT, AC, NC, or WHtR. In the T2DM subgroup, a statistically significant but weak negative correlation was observed between MT cIMT and FLI. No other significant correlations were identified. Detailed results are presented in Table 5 .

**Table 5:** Correlation between MT-cIMT and metabolic variables in the general population and T2DM group

Variable	General population (n = 114)	p-value	T2DM (n = 85)	p-value
	r		r	
FLI	–0.1483	0.1629	–0.2547	0.0375
CAP	–0.1009	0.2986	–0.1243	0.2658
GGT	0.0015	0.9875	0.1021	0.3740
AC	–0.0462	0.6350	–0.0444	0.6956
NC	–0.0657	0.4970	–0.0229	0.8389
WHtR	0.1200	0.2138	0.0563	0.6177

AC: abdominal circumference; CAP: controlled attenuation parameter; FLI: fatty liver index; GGT: gamma-glutamyl transferase; NC: neck circumference; T2DM: type 2 diabetes mellitus; WHtR: waist-to-height ratio; r: Pearson correlation coefficient; p-value: significance level (p < 0.05 considered statistically significant).

## Discussion

CVD affects nearly half of individuals with MASLD, with carotid atherosclerosis reported in approximately 35% of participants with steatotic liver disease.^24^

In this prospective study, the relationship between MASLD and subclinical atherosclerosis was evaluated using cIMT, VA, and the presence of carotid atherosclerotic plaques as predictive markers. These data are scarce in the Brazilian literature. Moreover, to our knowledge, this is the first study conducted in a Brazilian population with MASLD to assess VA based on cIMT.

Among the 114 participants at risk for MASLD, recruited from an endocrinology outpatient clinic, 86.8% were diagnosed with liver steatosis and 27.2% with liver fibrosis. In the general population, the prevalence of steatosis ranges from 30% to 40%, while liver fibrosis affects approximately 5% to 10%.^2^The higher prevalence observed in the present study may be explained by the sample characteristics, which included a large proportion of participants with T2DM (75.4%), a known risk factor for both steatosis and fibrosis.^1^Additionally, a substantial portion of the study population consisted of postmenopausal women, a group in which the relationship between liver fibrosis and CVD remains underexplored.^25^

The severity of MASLD is associated with an increased risk of atherosclerosis and mortality,^12^and patients with T2DM may represent a particularly vulnerable subgroup.^5^However, several risk factors are shared between MASLD and atherosclerosis — such as obesity and MetS — which may act as confounders. In this study, no significant differences were observed in such potential confounders when comparing participants with and without liver fibrosis, including in the T2DM subgroup analysis. The groups were similar in terms of obesity prevalence and other anthropometric measures of adiposity, except for HC, which was higher in diabetic participants with fibrosis. No differences were found regarding age, sex, smoking status, laboratory parameters, or statin use.

GGT is an enzyme involved in the glutathione system, which serves as a key antioxidant mechanism in the body. Elevated GGT levels in individuals with fibrosis may indicate the presence of oxidative stress. Similarly, cardiovascular risk is linked to oxidative stress and chronic inflammation — both of which are reflected by increased GGT levels. Thus, GGT may serve as a readily available and cost-effective biomarker that is elevated in both liver fibrosis and cardiovascular disease.^26 , 27^

The present study did not demonstrate a significant association between liver fibrosis and atherosclerotic plaques detected by carotid ultrasound, consistent with findings from other studies.^28 - 30^In contrast, some previous studies have reported such an association. These discrepancies may be explained by differences in methodology as well as clinical, ethnic, and demographic characteristics of the study populations, besides the limited sample size.^31 - 33^

In our study, most participants were women, many of whom were using statins and had glycated hemoglobin levels close to target values. These factors were similar between the fibrosis and non-fibrosis groups. Moreover, data on the Brazilian population are scarce, as most studies have focused on Asian populations.^2^

Elevated cIMT is an independent risk factor for cardiovascular and cerebrovascular events.^8^In our population, cIMT was assessed and found to be higher in participants with fibrosis compared to those without fibrosis, particularly in the subgroup with T2DM.

Some authors have found no association between MASLD and increased cIMT, including studies conducted in participants with T2DM.^30 , 33 , 34^Conversely, other studies have demonstrated a positive association between MASLD and increased cIMT.^31 , 32 , 35^

Carotid ultrasound can be used to assess VA through parameters such as cIMT, carotid distensibility, and pulse wave velocity (PWV). Each of these measurements provides important information on the functional and structural condition of the arteries, contributing to a more comprehensive evaluation of vascular aging. In this study, VA was higher than CA in participants with liver fibrosis, both in the overall sample and in the T2DM subgroup — with a statistically significant difference in the latter ( Table 4 ). Participants with T2DM and fibrosis had a VA that was nine years greater than their CA. This finding suggests a possible association between liver fibrosis and accelerated cardiovascular aging in individuals with T2DM. A separate longitudinal study conducted in Brazil evaluated 291 patients with T2DM and MASLD, using carotid-femoral PWV to assess VA. The study found that high or increasing aortic stiffness predicted the development of advanced liver fibrosis.^36^Additionally, another Brazilian study showed elevated VA in individuals with type 1 diabetes (T1D).^37^Taken together, these findings suggest that diabetes mellitus contributes to increased VA, reflecting higher cardiovascular risk and endothelial dysfunction.^37^

Zhou et al. (2018) demonstrated that MASLD is independently associated with a higher prevalence of CVD in patients with T2DM and proposed MASLD as an additional risk factor for CVD in this population.^38^

A recent meta-analysis of 7,951 participants at risk for MASLD showed that the prevalence of carotid atherosclerosis varied by geographic region, being highest in Europe (45%), followed by North America (41%), Asia (36%), and lowest in the Middle East (19%).^24^These findings underscore the lack of studies focusing on the Latin American population.

Sinn et al. (2016) observed that among individuals with MASLD, the frequency of carotid plaques was higher in those with a lower average age.^39^The association between MASLD and carotid plaque was more pronounced in younger adults than in older ones. However, they noted that the sensitivity and specificity of MASLD for predicting carotid plaque are limited. Despite this, the authors did not support routine MASLD screening in the general population solely for cardiovascular risk assessment.^39^Shao et al. (2020) proposed that age and BMI may serve as predictors of cardiovascular risk in individuals with MASLD.^40^

Although steatosis quantification by CAP did not differ significantly between participants with and without fibrosis — including in the T2DM subgroup — the highest CAP values were observed in those with fibrosis, suggesting more advanced disease. Eddowes et al. (2019) demonstrated that while CAP has limited discriminatory power for distinguishing between grade 2 and grade 3 steatosis (moderate vs. severe), it shows good accuracy for the diagnosis of steatosis compared with liver biopsy.^21^Therefore, steatosis quantification alone is not considered a reliable indicator of disease severity. Nevertheless, our findings may indicate that, in a population at risk for MASLD, elevated CAP values could be associated with more serious steatotic disease.

Among the limitations of this study is its cross-sectional design, which precludes the establishment of causal relationships between MASLD and increased cIMT. The regional specificity of the sample also limits the generalizability of the findings to the broader Brazilian and global populations. In addition, the relatively small sample size and convenience sampling from a tertiary care hospital may introduce selection bias, potentially overrepresenting more severe cases and limiting applicability to the general population. On the other hand, our study has a prospective design, and the exams were performed by a single experienced examiner. Despite these limitations, the study has strengths. It employed a prospective design, and all imaging assessments were performed by the same experienced examiner, ensuring methodological consistency and reliability.

The diagnosis of liver disease and its progression (steatosis and fibrosis) in this study was based on liver ultrasound and elastography, rather than liver biopsy — the current gold standard. However, the use of an invasive procedure such as biopsy on a large scale is impractical and poses unacceptable risk to patients.

In this study, conducted in the Brazilian population of the metropolitan region of Niterói, participants with moderate to advanced liver fibrosis associated with MASLD exhibited higher cIMT and VA, with statistical significance observed in the T2DM subgroup.

Despite ongoing debate in the international literature, MASLD remains an underestimated and independent risk factor for atherosclerotic cardiovascular disease. It shares several underlying mechanisms with CVD, including low-grade systemic inflammation, endothelial dysfunction, and insulin resistance.^41 - 44^

Because of regional variations in population characteristics, further studies are clearly needed to better assess the association between liver fibrosis due to MASLD and increased cIMT, VA, and the presence of atherosclerotic plaques.

In the context of global cardiovascular risk assessment in patients with T2DM, liver fibrosis due to MASLD may represent an important factor to consider. Further research is warranted to confirm this hypothesis.

## Conclusions

This study highlights the potential role of liver fibrosis due to MASLD — particularly in individuals with T2DM — as an intermediate marker of cardiovascular risk. Among Brazilian participants at risk for MASLD, those with T2DM and liver fibrosis demonstrated higher cIMT and VA, suggesting early vascular aging and elevated cardiovascular risk. These findings reinforce the importance of incorporating intermediate indicators, such as cIMT and VA, alongside traditional cardiovascular risk calculators in the comprehensive assessment of individuals with MASLD.

Data Availability

All datasets supporting the results of this study are available upon request from the corresponding author. The dataset is not publicly available as in accordance with Brazilian ethical standards for human research, we cannot make the complete clinical dataset publicly available commitment to protecting participants’ privacy and confidentiality. This approach follows both the Brazilian National Health Council Resolution 466/2012 and the General Data Protection Law (LGPD - Law 13.709/2018). However, we strongly believe in scientific transparency and are committed to responsible data sharing. The pseudonymized datasets underlying the findings presented in this article will be available upon reasonable request to the corresponding author(s), subject to approval from the original Ethics Research Committee that authorized our study.
